# SLC25 in intestinal inflammation: mechanisms, pathological roles, and therapeutic implications

**DOI:** 10.3389/fimmu.2026.1915452

**Published:** 2026-09-04

**Authors:** Juan Xu, Zan Zuo, Pei Wang, Tian He, Linting Xun, Ying An, Mei Luo, Jialong Qi, Ping Wan

**Affiliations:** 1 Department of Gastroenterology, the First People’s Hospital of Yunnan Province, School of Medicine, Kunming University of Science and Technology, Kunming, Yunnan, China; 2 First People’s Hospital of Yunnan Province, Yunnan Province Clinical Medical Center for Digestive System Diseases, Kunming, Yunnan, China; 3 The First People’s Hospital of Yunnan Province, Yunnan Clinical Medical Center for Blood Disease and Thrombosis Prevention and Control, Kunming, Yunnan, China

**Keywords:** immunometabolism, inflammatory bowel disease, intestinal immunity, macrophage polarization, mitochondrial dysfunction, mucosal inflammation, SLC25 transporters

## Abstract

Inflammatory bowel diseases (IBD) represent a spectrum of chronic gastrointestinal disorders fueled by a complex multifactorial interplay, where mitochondrial dysfunction has emerged as a critical contributor. The SLC25 family of mitochondrial transporters mediates the exchange of essential metabolites across the mitochondrial inner membrane, serving as pivotal regulators of cellular energy homeostasis in both the intestinal epithelium and immune cells. Accumulating evidence indicates that dysregulation of SLC25-dependent metabolic transport disrupts the crosstalk between cellular metabolism and immune signaling, driving chronic intestinal inflammation. This review comprehensively examines the pathological roles of SLC25 proteins across three interconnected dimensions: epithelial barrier disruption, immune dysregulation, and host-microbiota metabolic crosstalk. Specifically, we synthesize current findings on how altered SLC25 function compromises the intestinal barrier via oxidative stress and energy deficits, skews immune cell polarization toward pro-inflammatory states, and perpetuates a vicious cycle of microbial dysbiosis. Ultimately, this review provides a comprehensive theoretical basis for the central role of SLC25-mediated metabolic reprogramming in IBD pathogenesis, and establishes a critical mechanistic foundation for the development of novel metabolism-targeted therapeutics and diagnostic biomarkers.

## 1 Introduction

Inflammatory bowel diseases (IBD), encompassing Crohn’s disease (CD) and ulcerative colitis (UC), are chronic and debilitating conditions affecting the gastrointestinal tract (GI tract) (1). The pathogenesis of IBD is driven by a complex convergence of genetic predispositions, environmental triggers, microbial dysbiosis, and immune dysregulation (2–5). The incidence and prevalence of IBD are steadily rising worldwide, placing a significant burden on healthcare systems. Despite substantial advances in biologic and small-molecule therapies, a considerable proportion of patients still fail to achieve sustained remission, underscoring the critical need for a deeper mechanistic understanding of IBD pathogenesis (6, 7). Notably, mitochondrial dysfunction emerges as a key driver of IBD, promoting excessive reactive oxygen species (ROS) production, bioenergetics failure, and epithelial damage, which collectively amplify intestinal inflammation (8–10).

The Solute Carrier (SLC) superfamily encompasses hundreds of membrane transport proteins that mediate the exchange of diverse substances, including nutrients, ions, and metabolites, across cellular membranes, serving as the critical “metabolic gates” of cells (11). In the gastrointestinal tract, the broader SLC family plays a fundamental role in maintaining mucosal homeostasis, and its dysregulation is deeply implicated in intestinal inflammation and IBD. For instance, specific plasma membrane SLC members such as SLC7A2 regulate L-arginine availability, modulating macrophage polarization and protecting against colitis-associated carcinogenesis (12). Furthermore, transporters like SLC6A14 have been shown to exacerbate ulcerative colitis by facilitating NLRP3 inflammasome-mediated pyroptosis, while organic cation transporters (OCTNs/SLC22 family) mediate the transport of gut microbiota by-products and anti-inflammatory compounds, influencing Crohn’s disease susceptibility (13). These broad impacts underscore that SLC-mediated metabolic transport is intrinsically linked to the cascade of intestinal immune responses.

While plasma membrane SLCs manage extracellular nutrient uptake, cellular metabolism is ultimately centralized within the mitochondria, where metabolic reprogramming dictates immune cell activation and epithelial barrier integrity (2). This makes the mitochondrial sub-branch of the SLC superfamily—the SLC25 family—a crucial upstream regulator of mitochondrial function and metabolic reprogramming. By strictly controlling the flux of critical metabolites (such as ATP, TCA cycle intermediates, and redox buffers) across the inner mitochondrial membrane, SLC25 transporters directly govern mitochondrial bioenergetics and retrograde signaling to the nucleus (2, 14). Therefore, dysfunctional SLC25 transporters are not merely secondary bystanders to inflammation, but primary initiators of the energy collapse and oxidative stress that drive IBD pathology (15).

Accumulating evidence highlights specific, high-resolution mechanisms by which SLC25 dysfunction drives mucosal inflammation in IBD, dictating precise inflammatory outcomes through targeted substrate transport (16). For example, in the innate immune compartment, the upregulation of the mitochondrial citrate carrier SLC25A1 exports citrate to the cytosol, directly fueling acetyl-CoA production for pro-inflammatory lipid synthesis and locking macrophages into a hyper-inflammatory M1 phenotype (5, 17). Furthermore, the depletion of the mitochondrial glutathione carrier SLC25A39 disrupts mitochondrial redox homeostasis, leading to excessive ROS accumulation that directly triggers RIPK1/RIPK3-mediated necroptosis and epithelial barrier collapse (18). Additionally, members like the oxodicarboxylate carrier SLC25A21 specifically regulate the downstream expression of potent pro-inflammatory chemokines such as CXCL8, serving as a direct link between metabolite transport and neutrophil-driven mucosal damage (19). These targeted examples demonstrate that SLC25-mediated metabolic reprogramming is deeply integrated into the specific pathological cascades of intestinal inflammation.

This review comprehensively examines the role of SLC25 mitochondrial transporters in intestinal inflammation. We first outline the SLC25 family’s structural features and substrate specificities, and then we focus on how SLC25-dependent metabolite transport regulates the crosstalk between cellular metabolism and immune signaling in IBD. We discuss emerging evidence that SLC25 dysfunction disrupts energy/redox homeostasis and reprograms immune-epithelial responses, driving chronic inflammation. By integrating current findings, this review aims to highlight SLC25-dependent metabolic–immune circuits as central to IBD pathogenesis and propose them as promising therapeutic targets or adjuvant treatment means.

## 2 SLC25-mediated metabolic reprogramming in intestinal inflammation

**Figure 1:**
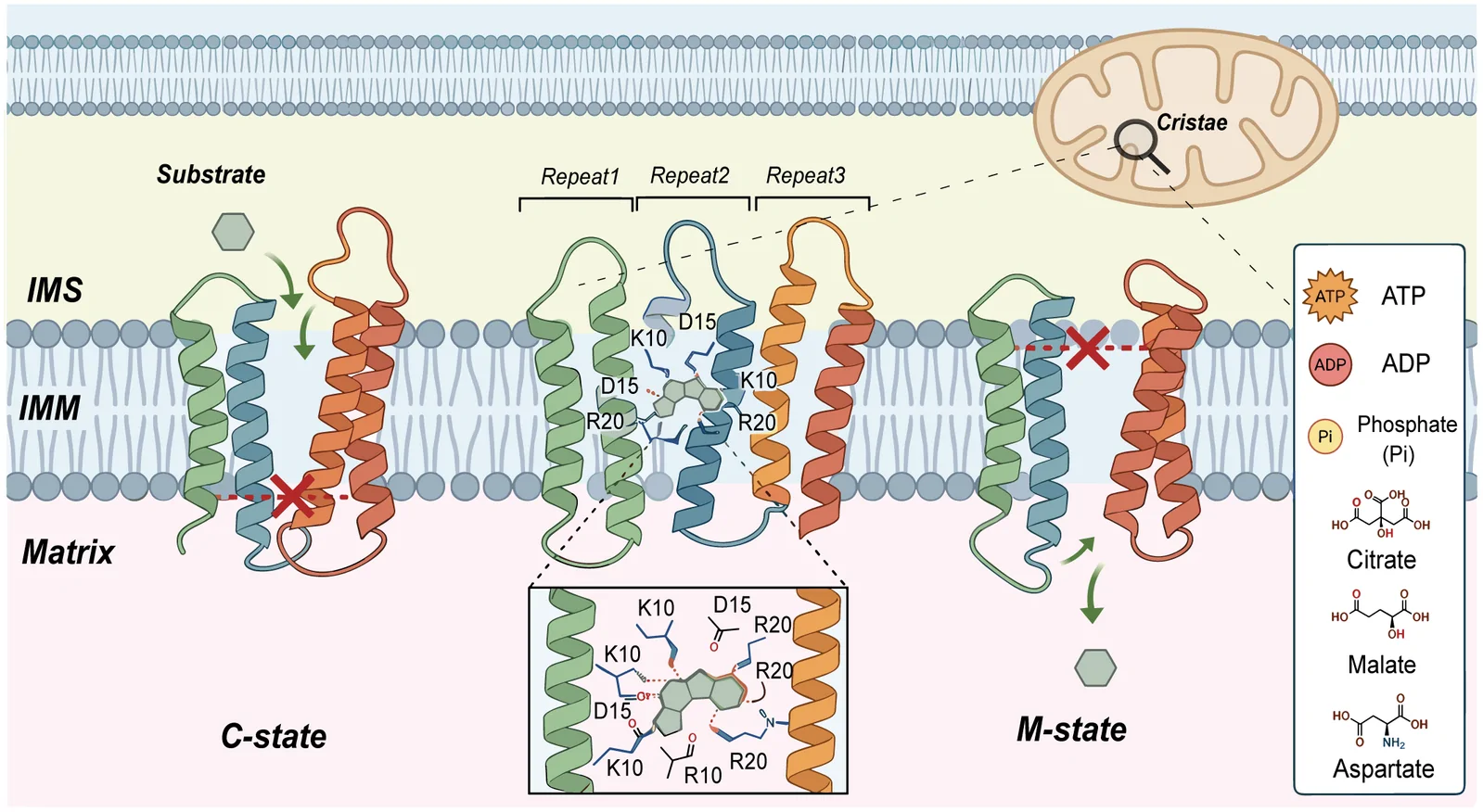
Conformational cycle of the mitochondrial carrier. The diagram illustrates the alternating access model across the IMM. Substrate translocation is driven by structural transitions from the IMS-facing C-state to the matrix-facing M-state. The inset details the substrate-binding pocket, revealing essential coordinating residues within the homologous repeats.

The SLC25 family functions as a critical interface connecting mitochondrial metabolism to cellular fate in the intestinal mucosa (20). To comprehend their pathological role in IBD, it is essential to first recognize their unique structural architecture (20). The human SLC25 carriers share a highly conserved pseudo-threefold symmetric architecture, primarily defined by three homologous tandem repeat domains (21). This framework yields six transmembrane α-helices that enclose a singular central cavity serving as the primary substrate-binding site (22). By utilizing an alternating-access “ping-pong” kinetic mechanism, these proteins transition between cytoplasmic-open (C-state) and matrix-open (M-state) conformations, strictly gating the flux of essential metabolites across the inner mitochondrial membrane (IMM) (8, 23) (**Figure 1**).

Under physiological conditions, this precise metabolic exchange maintains mucosal barrier integrity and immune tolerance (20). However, in the highly oxidative and inflammatory microenvironment characteristic of IBD, this delicate structural dynamic is severely compromised. Excessive reactive oxygen species (ROS) and lipid peroxidation byproducts induce irreversible post-translational modifications, such as the oxidation of critical cysteine residues or carbonylation of basic amino acids within the central substrate-binding cavity (24, 25). These biochemical modifications structurally uncouple the alternating-access mechanism, essentially locking the carriers in a non-functional C-state and creating a rigid transport bottleneck (26). Dysfunctional SLC25 transporters drive a core pathogenic shift: the aberrant flux of energy carriers, carboxylic acids, and redox buffers tightly links defective mitochondrial bioenergetics to widespread mucosal inflammation (6). Rather than merely being secondary bystanders, these structural and transport dysfunctions actively reprogram cellular metabolic networks, directly precipitating epithelial barrier collapse and skewing immune cell polarization in the gut microenvironment (17, 27).

Recent mechanistic insights into the pathological roles of SLC25 transporters in mucosal immunity and inflammation have been firmly established through diverse and high-resolution experimental models, ranging from genetic knockouts to targeted pharmacological inhibition (20). Clinical transcriptomic analyses of colonic cohorts have revealed that the aberrant expression of specific carriers, such as SLC25A5 and SLC25A6, significantly correlates with local CD8+ T cell depletion and skewed pro-inflammatory M1 macrophage polarization in the mucosal microenvironment (17, 20). In parallel, utilizing *in vitro* combinatorial CRISPR screens and targeted genetic deletion models, researchers have demonstrated that the loss of the glutathione carrier SLC25A39 disrupts mitochondrial redox homeostasis, driving massive ROS accumulation and subsequent epithelial cell death (18, 28). Furthermore, targeted pharmacological inhibition models have shown that blocking the oxoglutarate carrier SLC25A11 directly induces TCA metabolite accumulation and lipid peroxidation, accelerating ferroptosis-mediated tissue damage (27). At the systemic *in vivo* level, genetic loss-of-function mutant mouse models (such as SlC25A46 knockout mice) provide direct experimental evidence that SLC25 deficiency severely impairs adaptive immune cell development and triggers distinct lymphoid pathological phenotypes (29). Finally, advanced biochemical liposome reconstitution assays have definitively proven that host carriers like SLC25A14 and SLC25A30 actively transport and detoxify microbiota-derived hydrogen sulfide breakdown products, establishing a concrete biochemical foundation for their protective functions at the host-microbiota interface (14).

### 2.1 Epithelial barrier disruption: the SLC25 metabolic regulatory network

#### 2.1.1 Energy deficits and tight junction collapse

The structural and functional integrity of the intestinal epithelial barrier relies on a continuous mitochondrial energy supply. Within this context, the ATP/ADP exchange mediated by mitochondrial adenine nucleotide translocators (ANTs/SLC25A4) serves as a cornerstone of intestinal homeostasis (21). In intestinal epithelial cells, efficient ATP export from the mitochondria to the cytosol is a strict prerequisite for maintaining actin cytoskeleton dynamics and the phosphorylation-dependent assembly of tight junction proteins, such as ZO-1 and occludin (30).

When the expression or function of these nucleotide carriers is compromised, the resulting bioenergetic deficit precipitates a multi-layered structural collapse of the epithelial barrier. First, insufficient ATP export directly triggers cellular energy-sensing checkpoints, notably the rapid hyperactivation of AMP-activated protein kinase (AMPK) (31). Chronic AMPK activation in the energy-starved epithelium alters actin cytoskeleton dynamics by modulating myosin light chain kinase (MLCK) activity, which mechanically induces the caveolin-mediated endocytosis and structural disassembly of critical tight junction proteins, such as ZO-1 and occludin (32, 33). Second, this energy failure heavily impacts the mucosal biochemical shield. The maintenance of the protective mucus layer requires highly energy-intensive mucin synthesis and secretion by goblet cells (34). SLC25-driven mitochondrial metabolic failure significantly impairs these secretory functions, directly leading to mucus layer thinning and leaving the underlying epithelium highly vulnerable to luminal stress (35).

#### 2.1.2 Secondary mitochondrial dysfunction via NAD^+^ depletion

Beyond physical barrier disruption, nucleotide carrier dysfunction fundamentally alters the intracellular metabolic signaling landscape. For instance, defective SLC25A4 transport acutely alters the intracellular adenylate pool ratio (ATP/ADP). This energy imbalance translates metabolic stress into early pro-inflammatory signals, thereby sensitizing the epithelium to mucosal injury (23, 30). Furthermore, compromised mitochondrial transport of other essential nucleotide cofactors, such as NAD+, exacerbates this metabolic stress (36). The functional depletion of the mitochondrial NAD^+^ transporter (SLC25A51) drives downstream secondary mitochondrial dysfunction, creating a vicious cycle of persistent barrier failure and chronic mucosal inflammation (37).

#### 2.1.3 Redox imbalance and epithelial cell death

**Figure 2:**
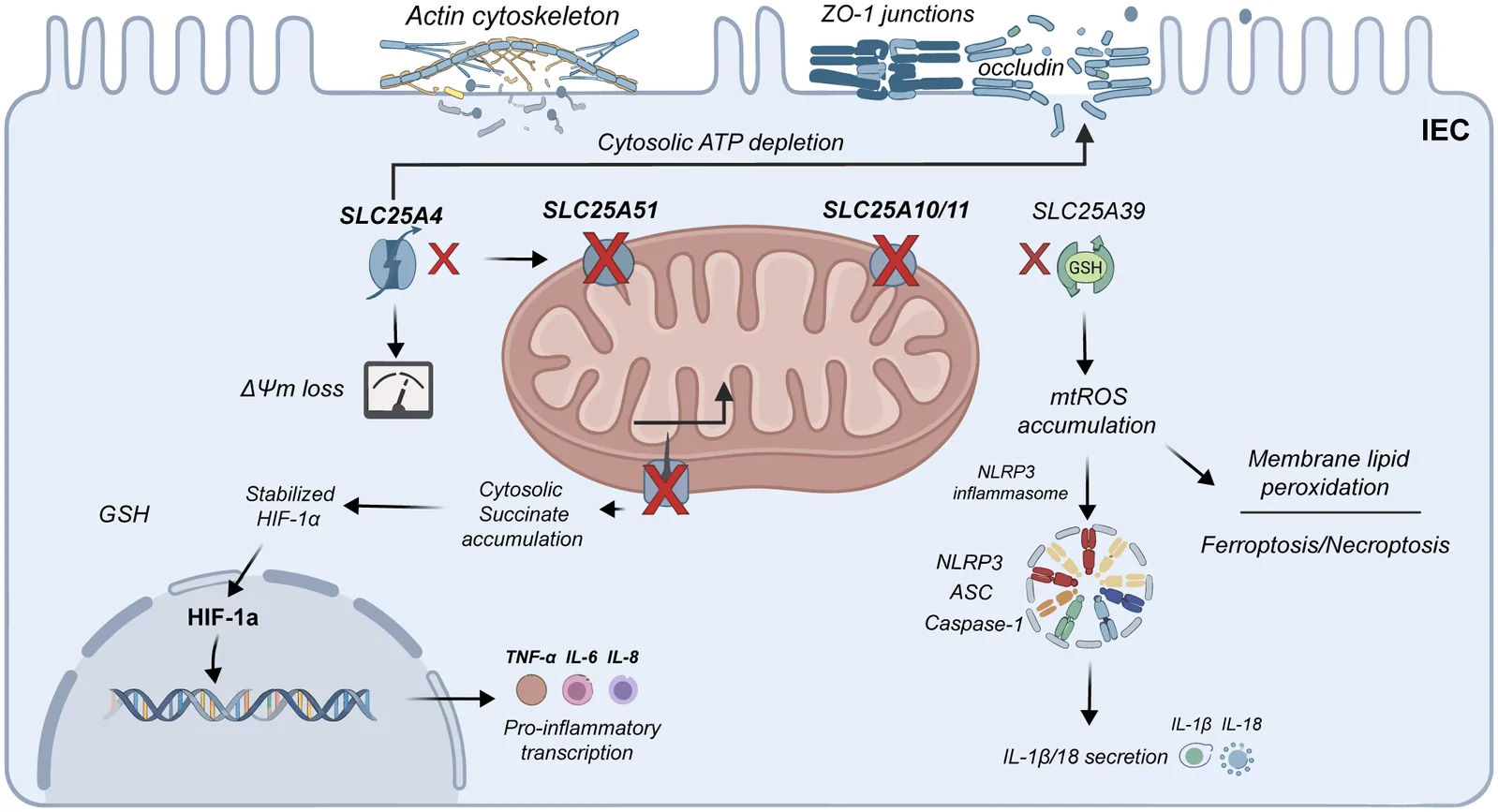
SLC25 carrier dysfunction drives inflammation and cell death in intestinal epithelial cells (IECs). Barrier Disruption: SLC25A4 impairment causes cytosolic ATP depletion and $\text{Δ}\psi_{m}$ loss, directly disrupting the actin cytoskeleton and tight junctions (ZO-1, occludin). Pro-inflammatory Transcription: Defective SLC25 transport (e.g., via SLC25A51, SLC25A10/11) leads to cytosolic succinate accumulation, stabilizing HIF-α to induce TNF-α, IL-6, and IL-8 expression. mtROS and Cell Death: Loss of SLC25A39-mediated GSH import results in mitochondrial ROS (mtROS) accumulation. This activates the NLRP3 inflammasome (driving IL-1β and IL-18 secretion) and induces membrane lipid peroxidation, culminating in ferroptosis or necroptosis.

In addition to energy and nucleotide cofactors, epithelial barrier maintenance heavily depends on SLC25-mediated redox buffering and TCA cycle flux. The transport of glutathione (GSH) into the mitochondria by SLC25A39 establishes a critical antioxidant defense axis (38, 39). Pathological suppression of SLC25A39 in the inflamed epithelium deprives mitochondria of GSH, which directly cripples the activity of Glutathione Peroxidase 4 (GPX4) (38). As a master regulator of ferroptosis, GPX4 strictly relies on GSH to neutralize toxic lipid hydroperoxides. The severe antioxidant depletion caused by SLC25A39 dysfunction leaves these lipid peroxides unchecked, effectively driving the epithelial cell fate toward ferroptotic cell death (40). Concurrently, to counteract this massive oxidative stress, the cellular defense machinery attempts to activate the Nrf2/Heme Oxygenase-1 (HO-1) signaling axis (41). While HO-1 upregulation serves as a critical cytoprotective response to degrade pro-oxidant heme and generate antioxidant biliverdin, the profound metabolic collapse initiated by SLC25 dysfunction often overwhelms this HO-1 compensatory mechanism. Concurrently, the transport of TCA cycle intermediates by carriers such as SLC25A11 (oxoglutarate/malate) and SLC25A10 (dicarboxylate) is vital for metabolic flexibility (42). The functional impairment of SLC25A11 triggers the aberrant accumulation of cytosolic TCA metabolites, acting as pseudohypoxic signals that sustain early inflammatory transcription (27). Ultimately, the spatiotemporal dysregulation across this integrated SLC25 network propels massive ROS accumulation and widespread lytic cell death, leading to the complete physical collapse of the mucosal shield (**Figure 2**).

### 2.2 Immune dysregulation: TCA cycle intermediates and redox imbalance

In the chronically inflamed intestinal mucosa, cellular metabolic reprogramming dictates both the polarization of immune responses and the overall survival of the mucosal barrier. Within this landscape, TCA cycle intermediates—and the amino acids that anaplerotically fuel them—act as potent signaling molecules, while the inevitable byproduct of this metabolic overdrive, reactive oxygen species (ROS), places immense strain on antioxidant defenses (43, 44). The SLC25 family rigidly controls the compartmentalization of these critical metabolites and redox buffers, directly coupling mitochondrial metabolic flux to immune dysregulation and inflammatory cell death.

#### 2.2.1 Macrophage M1 polarization via citrate efflux

TCA Intermediates and Immune Polarization In the innate immune compartment, the functional polarization of intestinal macrophages is intimately regulated by the mitochondrial citrate carrier, SLC25A1. During IBD flare-ups, pro-inflammatory cytokines (such as TNF-α and IFN-γ) significantly upregulate mucosal SLC25A1 expression via NF-κB and STAT1 signaling pathways (45, 46). This upregulation facilitates the massive export of mitochondrial citrate into the cytosol, where it is cleaved to generate acetyl-CoA. This cytosolic acetyl-CoA pool serves a dual pathogenic purpose: it provides essential carbon donors for histone acetylation—epigenetically driving the transcription of pro-inflammatory genes—and simultaneously fuels the *de novo* synthesis of pro-inflammatory lipid mediators, such as arachidonic acid (47). Consequently, this SLC25A1-driven metabolic shift locks macrophages into a hyper-inflammatory M1 phenotype. Conversely, targeted blockade or knockdown of intestinal SLC25A1 effectively curtails the release of IL-1β and IL-6, blocks M1 polarization, and significantly alleviates neutrophil infiltration in experimental colitis models (48).

#### 2.2.2 T cell activation and glutaminolysis

In parallel, the adaptive immune response in IBD—characterized by a pathogenic skewing towards Th1/Th17 cells and a deficit in regulatory T cells (Tregs)—is intricately shaped by mitochondrial amino acid carriers that feed the TCA cycle (17, 20). Mitochondrial carriers such as SLC25A13 (aspartate) and SLC25A22 (glutamate) stringently control the availability of precursors vital for T cell activation and clonal expansion (47, 49). SLC25A13 regulates the cytosolic aspartate pool required for massive nucleotide biosynthesis during T cell proliferation, while SLC25A22 dictates glutaminolysis, an essential anaplerotic pathway that sustains the bioenergetic demands of effector T cells. Disruption or pathological hijacking of these transporters alters the metabolic fitness of T cell subsets, fueling the expansion of pathogenic Th17 cells and exacerbating the breakdown of mucosal immune tolerance (1, 2, 48, 49).

#### 2.2.3 Innate lymphoid cell metabolic plasticity

**Figure 3:**
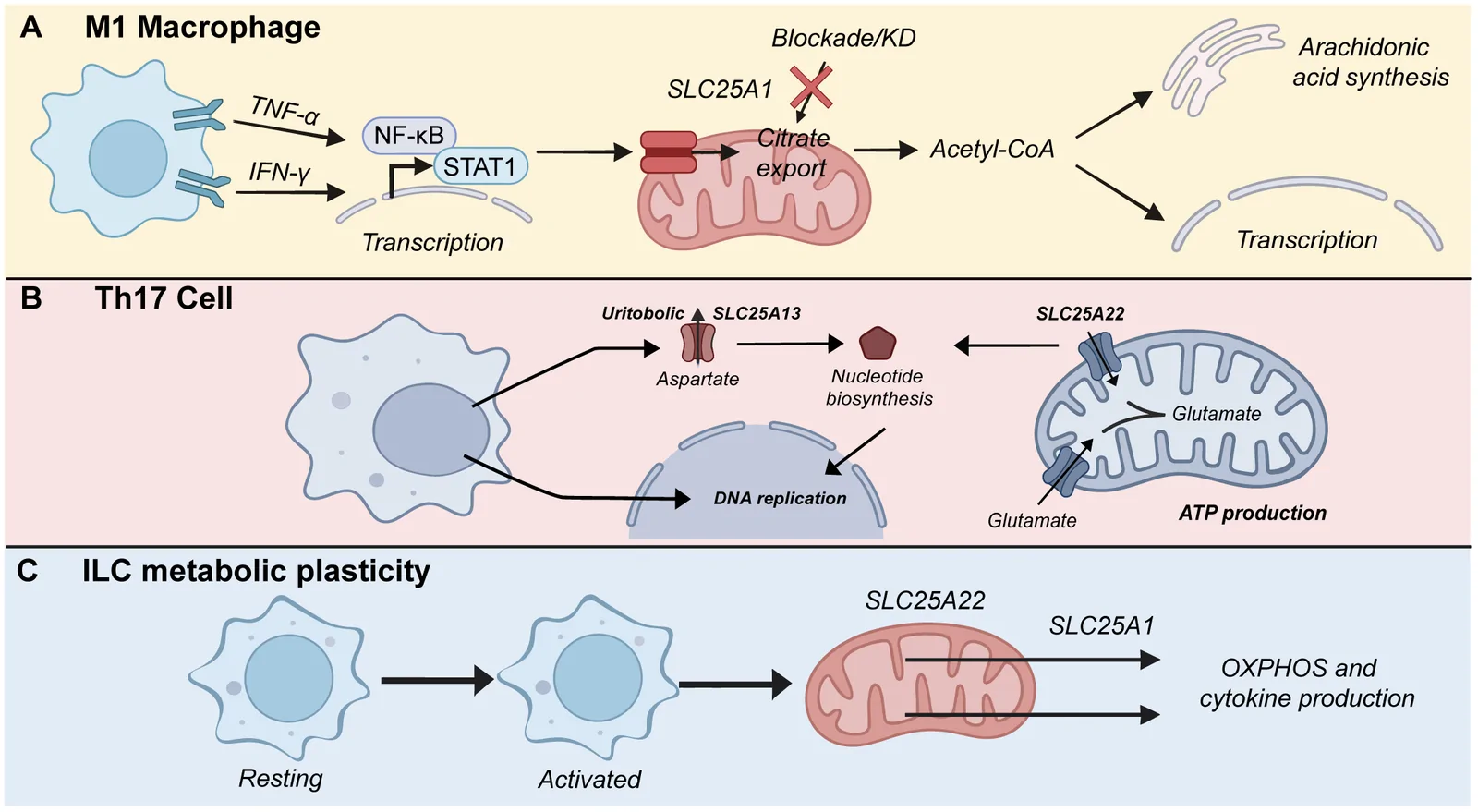
SLC25 mitochondrial carriers in immune cell metabolic reprogramming. **(A)** M1 Macrophages: TNF-α and IFN-γ upregulate *SLC25A1* via NF-κB and STAT1 to export mitochondrial citrate. The resulting cytosolic acetyl-CoA drives arachidonic acid synthesis and transcription, a process disrupted by *SLC25A1* blockade or knockdown.**(B)** Th17 Cells: SLC25A13-mediated aspartate export fuels nucleotide biosynthesis and DNA replication, while SLC25A22-dependent glutamate transport supports ATP production.**(C)** ILC Metabolic Plasticity: The transition of innate lymphoid cells (ILCs) to an activated state requires SLC25A22 and SLC25A1 to sustain oxidative phosphorylation (OXPHOS) and cytokine production.

Beyond classical innate and adaptive immune cells, innate lymphoid cells (ILCs)—particularly group 3 ILCs (ILC3s)—play an indispensable role in mucosal immunity and intestinal inflammation (1). The functional plasticity of ILCs in the gut is highly metabolically driven. During IBD progression, the pathogenic transdifferentiation of protective ILC3s into IFN-γ-producing ILC1-like cells is orchestrated by profound mitochondrial metabolic reprogramming, heavily relying on shifts in oxidative phosphorylation (OXPHOS), lipid oxidation, and glutaminolysis (1). While the direct mapping of individual SLC25 functional networks specifically within ILC subsets remains an emerging research frontier, their strict dependence on mitochondrial metabolite fluxes is undeniable (2, 50). The energy-intensive cytokine production and phenotypic shifts in ILCs inevitably require the precise import of amino acids (via carriers like SLC25A22) and the export of intermediates for lipid synthesis (via SLC25A1) (48, 49, 51). Consequently, SLC25-mediated metabolic gating is strongly implicated in governing ILC plasticity, presenting a critical uncharted territory for future immunometabolism research in IBD (**Figure 3**).

#### 2.2.4 Redox imbalance and immune cell fate

The profound metabolic reprogramming that fuels mucosal immune hyperactivation comes at a severe biochemical cost. In the relentless inflammatory milieu, highly active immune cells (such as M1 macrophages and effector T cells) generate massive amounts of reactive oxygen species (ROS) (52, 53). To sustain their survival and effector functions, these cells rely heavily on the precise compartmentalization of redox buffers by specific SLC25 carriers (38). Glutathione (GSH), the principal mitochondrial antioxidant, depends on dedicated importers, notably SLC25A39 and SLC25A40, to neutralize toxic lipid peroxides (38).

Under pathological IBD conditions, a severe metabolic mismatch frequently occurs within the immune compartment. The expression of pro-oxidant, inflammation-driving transporters (such as the citrate carrier SLC25A1) is significantly upregulated, fueling ROS production and NF-κB activation (54). Conversely, critical antioxidant transporters (SLC25A39/A40) can be pathologically overwhelmed or suppressed (38). This coordinated depletion of mitochondrial antioxidant defenses leads to explosive intracellular ROS accumulation. In mucosal immune cells, this unchecked oxidative stress not only locks macrophages into a sustained hyper-inflammatory state but can also ultimately trigger activation-induced cell death (AICD) in mucosal lymphocytes (55). Consequently, the breakdown of SLC25-mediated redox homeostasis directly perpetuates the chronic inflammatory cycle and prevents the restoration of mucosal immune tolerance.

Furthermore, the dysregulation of pyrimidine nucleotide carriers, particularly SLC25A33, introduces another critical layer of immune activation (56). During mucosal inflammation, the pathological accumulation of pyrimidine transport mediated by SLC25A33 not only exacerbates mitochondrial ROS production but also triggers cytosolic mitochondrial DNA (mtDNA) leakage (6, 56, 57). This cytosolic mtDNA acts as a potent danger signal, hyper-activating the cGAS-STING innate immune sensing pathway to sustain chronic inflammatory responses (10).

## 3 SLC25 transporters at the host-microbiota interface

The intestinal mucosa operates as a dynamic ecosystem where the host’s immune system continuously senses and processes metabolic cues from the gut microbiota (58, 59). In IBD, microbial dysbiosis characteristically disrupts this mutualistic balance (58, 60). Emerging evidence reveals that members of the SLC25 family are not merely passive intracellular housekeepers, but function as active, bidirectional conduits at the host-microbe interface (2). They dictate how the host detoxifies harmful bacterial catabolites and, conversely, how invading pathobionts hijack host metabolism for their own survival (61–63).

### 3.1 Host detoxification of microbial metabolites and symbiotic sensing

A crucial but frequently overlooked facet of host-microbiota crosstalk is the active detoxification of bacterial catabolites by host mitochondrial transporters to maintain mucosal homeostasis (44, 59). In the hypoxic colonic lumen, sulfate-reducing bacteria (SRB)—such as Desulfovibrio piger—often bloom excessively during IBD flare-ups. These bacteria generate high concentrations of hydrogen sulfide (H2S), a potent mucosal toxin capable of inducing epithelial barrier collapse (44, 58).

To counter this microbial toxicity, the host relies on specific SLC25 members. Biochemical evidence demonstrates that host uncoupling proteins UCP5 (SLC25A14) and UCP6 (SLC25A30) selectively transport sulfur oxyanions (such as thiosulfate and sulfite), which are the primary mitochondrial degradation derivatives of microbiota-derived H2S (61, 64, 65). By orchestrating the mitochondrial clearance and efflux of these sulfur metabolites, SLC25A14 and SLC25A30 establish a vital metabolic defense line, shielding the colonic epithelium from SRB-induced enterocyte poisoning and hyper-inflammatory damage (44, 61).

Concurrently, this host machinery intimately senses symbiotic inputs. Commensal-derived short-chain fatty acids (SCFAs, particularly butyrate) structurally sustain the expression of the carnitine carrier SLC25A20, safeguarding mitochondrial β-oxidation and maintaining the epithelial hypoxic niches essential for gut barrier integrity (58, 66, 67). Furthermore, symbiotic signals modulate host calcium-binding ATP-Mg/Pi carriers (e.g., SLC25A23), shaping the intracellular energetic currency to foster an anti-inflammatory milieu by promoting immunoregulatory M2 macrophage accumulation (17).

**Figure 4:**
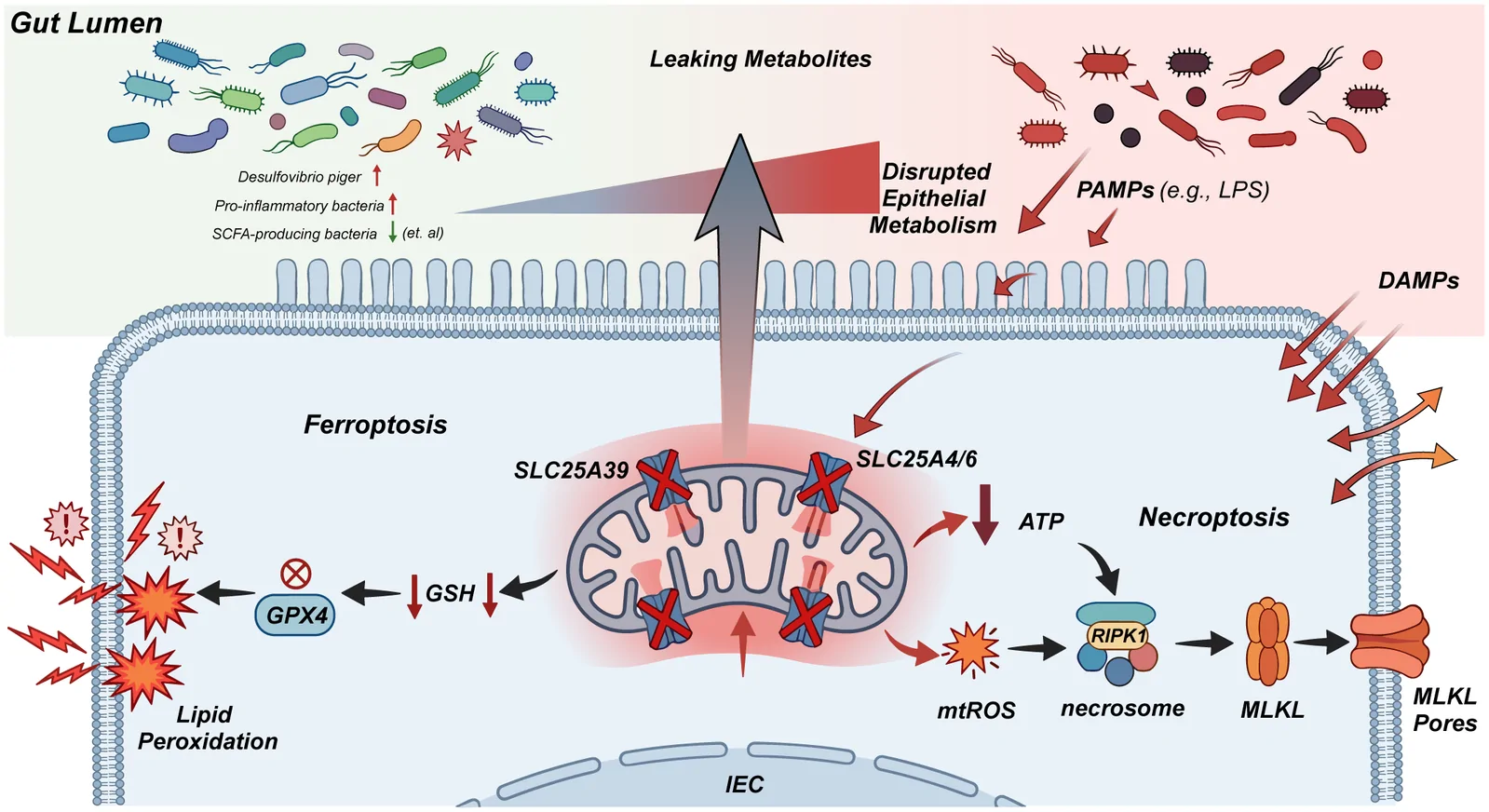
SLC25-driven mitochondrial dysfunction promotes lytic cell death in intestinal epithelial cells (IECs). Dysfunctional SLC25 transporters disrupt mitochondrial homeostasis, leading to the depletion of glutathione (GSH) and ATP. The decrease in GSH impairs GPX4 activity, triggering lethal lipid peroxidation and ferroptosis. Concurrently, severe ATP depletion and excessive mitochondrial ROS (mtROS) production promote the assembly of the RIPK1/RIPK3 necrosome, inducing MLKL oligomerization and pore formation, which characterizes necroptosis. This lytic cell death releases damage-associated molecular patterns (DAMPs) and facilitates the entry of luminal antigens, further exacerbating mucosal inflammation and barrier damage.

However, when microbial dysbiosis escalates—characterized by the overgrowth of pathobionts such as *Desulfovibrio piger* and a sharp decline in SCFA-producing bacteria—the excessive mucosal influx of pathogen-associated molecular patterns (PAMPs, such as LPS) completely overwhelms the SLC25-mediated metabolic defense (59). This host-microbiota metabolic crash precipitates catastrophic lytic cell death in intestinal epithelial cells (4). Specifically, the disruption of SLC25A39 severely depletes mitochondrial GSH, effectively crippling GPX4 activity and triggering lethal lipid peroxidation (ferroptosis) (38, 40). While the host epithelium attempts to counteract this massive oxidative stress by activating the cytoprotective HO-1 signaling axis, the profound metabolic collapse initiated by SLC25 dysfunction ultimately overwhelms this HO-1 compensatory mechanism. Concurrently, the failure of nucleotide translocators (SLC25A4/6) causes profound ATP depletion and rampant mtROS production, directly driving the assembly of the RIPK1/RIPK3 necrosome and subsequent MLKL pore formation (necroptosis) (41). The execution of these highly inflammatory cell death pathways releases massive amounts of damage-associated molecular patterns (DAMPs) back into the gut lumen, creating a vicious cycle of microbial dysbiosis and complete barrier destruction (28, 39) (**Figure 4**).

### 3.2 Microbial subversion, molecular mimicry, and nutritional immunity

The bidirectional interaction at the SLC25 interface extends beyond host-side responses; it incorporates sophisticated mechanisms of microbial evolutionary adaptation and molecular mimicry (62, 63). In the inflamed IBD gut, the strict anaerobic and nutrient-fluctuating landscape forces microorganisms to adapt (44). While specialized members of the human gut microbiota have evolved distinct mitochondrial carrier homologs to optimize their survival in this harsh environment (63). A premier example is the widespread gut eukaryotic commensal/pathogen Blastocystis hominis. Genomic and phylogenetic analyses indicate that Blastocystis expresses unique mitochondrial carrier homologs (GIC-1 and GIC-2) that exhibit high sequence conservation with the human dicarboxylate/tricarboxylate transporters SLC25A10 and SLC25A11 (63). These microbial transporters shuffle glycolytic intermediates across the organelle membrane, allowing the organism to aggressively rewire its energetic network to colonize the hostile IBD niche (63).

Parallelly, invasive pathobionts exploit the structural blueprints of the host’s SLC25 family to subvert mucosal defenses (62). Intracellular pathobionts secrete specialized effector proteins that exhibit striking molecular mimicry to human mitochondrial carriers. For instance, the bacterial effector protein Lpg1137 functions as a structural homolog of host SLC25 proteins (62). Upon delivery into the host cytoplasm, these bacterial mimics localize to the mitochondrial membrane, directly interfering with endogenous solute exchange, disrupting host energy production, and deliberately triggering lytic cell death cascades (62).

In a fascinating counter-evolutionary response, host cells deploy localized “nutritional immunity” managed by the transport network (44). To combat invasive pathobiont threats, host cells stringently downregulate the mitochondrial iron transporters Mitoferrin-1/2 (SLC25A37/SLC25A28), effectively starving intracellular pathogens of the critical iron supplies required for their replication cycle (68, 69). This intense “tug-of-war” over mitochondrial metabolite transport highlights the SLC25 family as a central battlefield in host-microbiota interactions during IBD pathogenesis (2, 44).

To systematically elucidate these multidimensional interactions, **Table 1** comprehensively summarizes the specific alterations of SLC25 family members across intestinal epithelial and diverse immune cell types, detailing their upstream triggers and profound impacts on IBD pathogenesis.

**Table 1:** Summary of SLC25-mediated metabolic reprogramming across distinct cell types in IBD pathogenesis.

Cell type	SLC25 member(s)	Expression/function changes in IBD	Triggering factors	Impact on cellular function	Contribution to IBD pathogenesis
Intestinal Epithelial Cells (Barrier Function)	SLC25A4, SLC25A6 (ANTs) (30, 36, 72)	SLC25A4 is downregulated; SLC25A6 is pathologically upregulated.	Metabolic stress, active inflammatory phase (correlated with CDAI).	Deficient cytosolic ATP export; disrupted actin dynamics and tight junction assembly (ZO-1, occludin); impaired mucin synthesis.	Increased paracellular permeability; mucus layer thinning; early pro-inflammatory signaling; luminal antigen translocation.
Intestinal Epithelial Cells (Redox & Cell Death)	SLC25A39, SLC25A40, SLC25A51 (28, 86)	Pathologically suppressed or lost.	Severe metabolic mismatch; chronic mucosal inflammation.	Depletion of mitochondrial glutathione (GSH) and NAD+; massive reactive oxygen species (ROS) accumulation; severe lipid peroxidation.	Execution of highly lytic cell death (necroptosis and ferroptosis); release of DAMPs; exacerbation of mucosal damage.
Macrophages (Innate Immunity)	SLC25A1 (Citrate Carrier) (45)	Significantly upregulated.	Pro-inflammatory cytokines (TNF-α, IFN-γ) via NF-κB and STAT1 signaling pathways.	Massive cytosolic efflux of mitochondrial citrate to generate acetyl-CoA; fuels histone acetylation and *de novo* arachidonic acid synthesis.	Locks macrophages into a hyper-inflammatory M1 phenotype; promotes massive release of IL-1β and IL-6; drives neutrophil infiltration.
T Cells (Adaptive Immunity)	SLC25A13 (Aspartate), SLC25A22 (Glutamate) (20, 49)	Hijacked/altered to meet hyper-metabolic demands.	Persistent antigenic stimulation; inflamed microenvironment.	Facilitates massive nucleotide biosynthesis (SLC25A13) and glutaminolysis (SLC25A22) to sustain bioenergetics.	Fuels the hyper-activation and clonal expansion of pathogenic Th17 cells; breaks mucosal immune tolerance.
Innate Lymphoid Cells (ILCs)	SLC25A1, SLC25A22 (and others) (1)	High dependence on precise metabolite gating for plasticity.	IBD progression; energy-intensive microenvironment.	Metabolic shifts relying on precise amino acid import and intermediate export for lipid synthesis.	Drives the pathogenic transdifferentiation of protective ILC3s into pro-inflammatory, IFN-γ-producing ILC1-like cells.

### 3.3 Genetic variants and pathological significance in IBD

While dynamic expression alterations of SLC25 transporters play a critical role in mucosal responses, specific genetic variants and mutations within these carrier genes further dictate the pathological susceptibility to IBD. Historically, severe genetic mutations (such as point mutations and deletions) in SLC25 genes have been extensively linked to systemic metabolic disorders characterized by profound organellar compartmentalization defects (70). However, emerging evidence indicates that genetic variants in the SLC25 family also carry specific pathological significance in mucosal immunity. For instance, genetic loss-of-function mutations in specific members, such as SLC25A46 (71), have been directly shown in mutant mouse models to trigger distinct lymphoid pathological phenotypes, severely impairing adaptive immune cell development and mucosal defense (29).

Furthermore, within the host-microbiota interactive axis, host genetic variations can critically compromise mucosal detoxification capabilities. If genetic variants impair the function of host sulfur-transporting uncoupling proteins (such as SLC25A14 and SLC25A30), the host’s biochemical capacity to clear toxic, microbiota-derived hydrogen sulfide is severely diminished (56). This host-side genetic vulnerability exacerbates enterocyte poisoning and accelerates epithelial barrier collapse during microbial dysbiosis. Collectively, these findings highlight that both genetic mutations and functional variants of SLC25 proteins are fundamentally tied to the pathogenic initiation and amplification of IBD.

## 4 Clinical applications and therapeutic value of SLC25 proteins

While the primary focus of this review is the mechanistic orchestration of IBD by the SLC25 family, these fundamental metabolic insights possess substantial translational value. Conventional IBD management strategies predominantly hinge on broad-spectrum immunosuppression (e.g., corticosteroids, monoclonal antibodies), which frequently entail substantial long-term risks, including secondary non-responsiveness or opportunistic infections (7, 72). Shifting the focus toward “metabolic remodeling” via SLC25 transporters offers a transformative clinical paradigm: resolving mucosal inflammation by directly repairing impaired mitochondrial bioenergetics and redox balance. This section briefly outlines the clinical potential of SLC25 proteins as dynamic diagnostic biomarkers and precision therapeutic targets.

### 4.1 Diagnostic and prognostic potential of SLC25 biomarkers

Current IBD diagnostics (such as CRP, fecal calprotectin, and endoscopy) predominantly reflect downstream tissue damage rather than the underlying cellular metabolic state. Emerging clinical cohort data suggest that the differential expression of SLC25 family proteins exhibits high disease specificity and can dynamically monitor mucosal metabolic-inflammatory evolution (72).

At the tissue level, clinical biopsies demonstrate that the expression of the ATP transporter SLC25A4 is negatively correlated with the Clinical Disease Activity Index (CDAI) and Mayo scores, whereas SLC25A6 is pathologically upregulated during active phases (72). Using the “SLC25A6/SLC25A4 expression ratio” serves as a predictive index to distinguish active IBD from remission (36). Furthermore, non-invasive detection of the pyrimidine carrier SLC25A33 (via fecal exfoliated cells and serum exosomes) shows significant promise for differentiating Ulcerative Colitis (UC) from Crohn’s Disease (CD) (36). Crucially, intestinal SLC25A33 levels drop significantly in patients who respond to biologics (e.g., Infliximab) or Fecal Microbiota Transplantation (FMT) within 2–4 weeks, highlighting its potential as an early, dynamic biomarker for therapeutic sensitivity (7, 36).

### 4.2 Pharmacological interventions targeting SLC25 and mitochondrial metabolism

Targeting the microbiota-metabolism-SLC25 interactive axis has shown definitive efficacy in *in vitro* cellular assays and *in vivo* colitis models. The pharmacological interventions currently under investigation can be categorized by their strategic approach to metabolic remodeling, as summarized in **Table 2**.

**Table 2:** Classification of candidate drugs and strategies targeting SLC25 proteins.

Drug category	Representative agent	Target & mechanism of action	Clinical/preclinical status
Targeted Small Molecule Inhibitors/Agonists	CTPI-2 (Specific Inhibitor) (17, 73)	SLC25A1 inhibition ↓ → Mitochondrial citrate efflux ↓ → M1 macrophage polarization & Pro-inflammatory lipid synthesis ↓	Preclinical (Significant reduction in colitis scores in animal models)
	SLC25A10 Agonists (63)	SLC25A10 activation ↑ → Malate-aspartate shuttle ↑ → Oxidative stress ↓ & Redox balance restored	Preclinical (*In vitro* and early animal studies)
	SLC25A33 Targeted Inhibitors (57, 72)	SLC25A33 inhibition ↓ → Cytosolic pyrimidine & mtDNA leakage ↓ → cGAS-STING pathway ↓ → mtROS ↓	Preclinical (Target validation phase)
Drug Repurposing	Metformin (75)	AMPK activation ↑/mTORC1 ↓ → SLC25A33 pathological overexpression ↓ → Intestinal energy homeostasis ↑	Clinical observation data available in IBD cohorts
	Mesalazine (5-ASA) (77) (87)	PPAR-γ activation ↑ → SLC25A20 (carnitine carrier) expression ↑ → Fatty acid β-oxidation ↑	Widely used clinically; novel mechanism confirmed
Natural Phytochemicals	Berberine (88)	SCFA-producing microbiota ↑ → SLC25 family function ↑ → Mitochondrial respiratory chain & Mucosal barrier repair ↑	Preclinical and early clinical exploration
	Quercetin/Resveratrol (18, 76)	SLC25A39 (GSH carrier) protection ↑ → Mitochondrial iron-sulfur cluster stability ↑ → Antioxidant defense ↑	Preclinical (Extensively validated)
	Ginsenosides (e.g., Rb1) (88)	Promotes energy metabolism in intestinal epithelial cells by upregulating core mitochondrial transporters to maintain ATP supply and inhibit apoptosis. Core mitochondrial transporters ↑ → ATP supply ↑ → IEC apoptosis ↓	Preclinical (Cellular and animal models)
Traditional Chinese Medicine (TCM) Formulas	ShenlingBaizhuSan (45) (77, 78)	SLC25A22 expression ↑ → Glutamate/proline transport ↑ → Mucosal immune homeostasis ↑	Widely used clinically; deep mechanistic studies
	HuangqinTang (79)	Pro-inflammatory bacteria & LPS load ↓ → Endotoxin interference on SLC25 family ↓	Preclinical and clinical adjuvant therapy
	WumeiWan (80)	Gut microecology reshaping ↑ → Mitochondrial structure repair ↑ → Microbial dysbiosis-mitochondrial damage cycle ↓	Widely used clinically

↑, indicates activation/increase; ↓, indicates inhibition/decrease; →, indicates downstream effect (or "leads to").

As detailed in **Table 2**, translating SLC25 mechanisms into therapeutic strategies provides diverse options. The development of specific small molecules, such as the SLC25A1 inhibitor CTPI-2 (17, 73) and targeted SLC25A33 blockers (57) (56), offers precision tools to explicitly halt immunometabolic dysregulation at defined checkpoints. Concurrently, the mechanistic repurposing of established clinical drugs—such as Mesalazine (5-ASA) rescuing SLC25A20-mediated β-oxidation (58, 74) and Metformin modulating SLC25A33 expression (75) provides immediate, safe avenues for clinical application. Furthermore, the holistic, multi-target properties of phytochemicals (e.g., Berberine, Resveratrol) (28, 76) and TCM formulas (e.g., Shenling Baizhu San, Wumei Wan) (77–80) demonstrate unique advantages in repairing the broader “microbiota-mitochondria” interconnected network.

### 4.3 Emerging nanomedicines and mitochondria-targeted delivery

**Figure 5:**
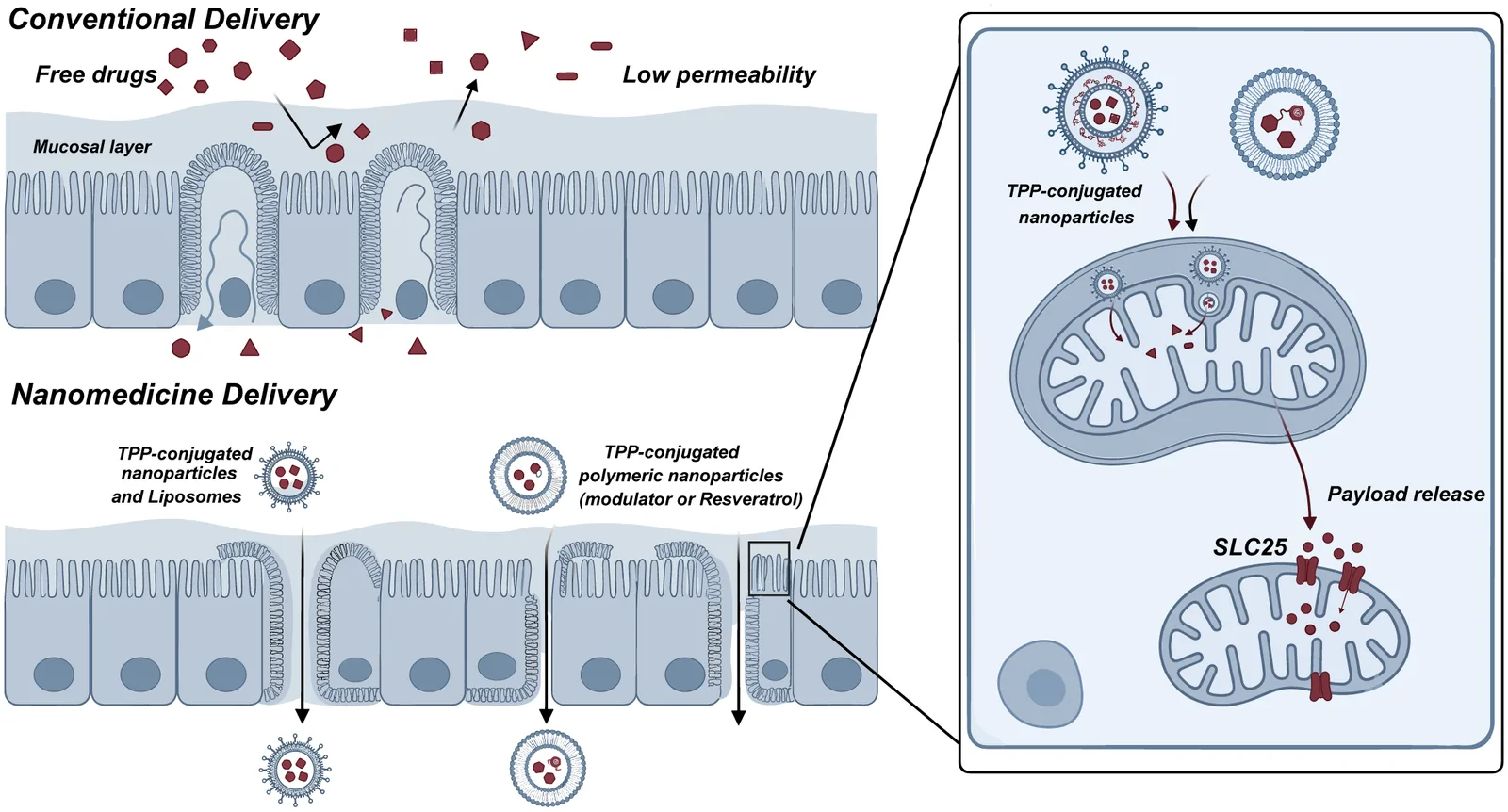
Mitochondria-targeted nanomedicine overcomes mucosal barriers to precisely deliver SLC25 modulators. Conventional delivery of free drug molecules is limited by poor intestinal mucosal permeability, resulting in inefficiency and off-target effects. Advanced nanomedicine strategies utilizing triphenylphosphonium (TPP)-conjugated polymeric nanoparticles and liposomes enhance epithelial barrier penetration. Upon cellular internalization, these engineered nanocarriers specifically target the mitochondria to release therapeutic payloads (e.g., CTPI-2 or Resveratrol) directly at SLC25 targets on the inner mitochondrial membrane, thereby maximizing local metabolic efficacy.

Despite the strong therapeutic potential of these metabolic modulators, clinical application is often limited by poor intestinal mucosal permeability and off-target systemic effects (81). To address this, emerging mitochondria-targeting nanomedicines, such as triphenylphosphonium (TPP) (82), conjugated liposomes and engineered polymeric nanoparticles—are being developed to encapsulate specific SLC25 modulators (e.g., CTPI-2 or Resveratrol) (83–85). These advanced delivery systems enable precise drug accumulation within the inflamed epithelium and facilitate direct internalization into the mitochondria, maximizing local metabolic efficacy while minimizing systemic toxicity (81). Ultimately, the convergence of SLC25-targeted pharmacology and precision nanomedicine represents a highly promising future trajectory for curative IBD therapeutics (**Figure 5**).

## 5 Challenges and future perspectives

Traditional IBD therapies rely heavily on generalized immunosuppression, whereas targeting SLC25 proteins offers a paradigm shift toward “metabolic remodeling.” However, the path to clinical translation faces several nuanced challenges that necessitate a more cautious, multi-dimensional perspective. Initially, the intrinsic functional redundancy inherent within the 53-member SLC25 family poses a substantial hurdle; compensatory upregulation of alternative transporters following the inhibition or deletion of a single member may mask its individual pathological significance, potentially explaining why the loss of certain family members does not universally trigger overt inflammation in all experimental settings. Second, while current research has successfully established a strong correlation between SLC25 dysfunction and IBD, the definitive establishment of a direct causal relationship remains elusive, as much of the existing evidence is derived from acute pharmacological perturbations rather than longitudinal studies that track metabolic shifts throughout the progression of the disease.

Furthermore, the structural homology shared across the SLC25 family represents a significant bottleneck for precision pharmacology; because these carriers are defined by a highly conserved pseudo-threefold symmetric architecture, the development of small-molecule modulators with sufficient isoform selectivity to avoid off-target systemic toxicity is exceedingly difficult. Finally, IBD is a complex, heterogeneous spectrum disease, yet current literature often fails to account for the distinct molecular and metabolic variances between Ulcerative Colitis (UC) and Crohn’s Disease (CD). To move beyond these limitations, future research must shift toward a precision medicine framework, utilizing single-cell spatial transcriptomics and metabolomics to map patient-specific SLC25 expression profiles and identifying unique allosteric binding sites via high-resolution structural biology to overcome the inherent risks of systemic mitochondrial interference. By addressing these gaps, we can transition from generalized metabolic observations to targeted, mechanism-driven therapeutics capable of achieving sustained clinical remission.

Ultimately, given the multifactorial nature of IBD, monotherapy targeting a single metabolic node may not suffice for sustained remission. The future of IBD treatment lies in the strategic convergence of SLC25-targeted metabolic correctors with existing clinical modalities, such as biologics or microecological therapies like Fecal Microbiota Transplantation (FMT). By synergistically addressing the root causes of both mitochondrial dysfunction and microbial dysbiosis, these integrated approaches hold immense translational promise, paving the way for curative, mechanism-driven regimens for IBD patients.

## 6 Conclusion

The SLC25 transporter family acts as an indispensable metabolic hub orchestrating the intricate cross-talk among mitochondrial bioenergetics, mucosal immunity, and host-microbiota communication in IBD. As elucidated in this review, the spatiotemporal dysregulation of these mitochondrial transporters is not merely an epiphenomenon of chronic inflammation but a fundamental driver of epithelial barrier collapse, immunometabolic skewing, and lytic cell death. By gating the flux of essential nucleotides, carboxylic acids, and redox buffers, these proteins establish a “metabolic checkpoint” that dictates the outcome of the intestinal inflammatory response.

The potential to transition these mechanistic insights into clinical practice is profound. Identifying SLC25 proteins as dynamic biomarkers offers a promising strategy for non-invasive disease monitoring and precision subtyping, moving beyond the limitations of traditional, non-specific inflammatory indices. Simultaneously, the emerging landscape of SLC25-targeted interventions ranging from precision small molecules and repurposed clinical agents to holistic TCM formulas and site-specific nanomedicine provides a multifaceted toolkit for metabolic remodeling.

The strategic integration of SLC25-targeted therapies with current clinical modalities holds the potential to transition IBD management from transient immunosuppression to long-term, mechanism-driven remission. Continued exploration into the “SLC25-mitochondrion-microbiota axis” will not only deepen our fundamental grasp of IBD pathogenesis but will ultimately establish a curative, patient-centric paradigm for the future of inflammatory disease therapeutics.
